# LTPLN: Automatic pavement distress detection

**DOI:** 10.1371/journal.pone.0309172

**Published:** 2024-10-10

**Authors:** Wen-Qing Huang, Liu Feng, Yuan-Lie He

**Affiliations:** 1 Guangdong ChengTech Tranffic&Technology Development Co., Ltd, Guangzhou, China; 2 Guangzhou Road Research Institute Co., Ltd, Guangzhou, China; 3 School of Computers, Guangdong University of Technology, Guangzhou, China; Shandong University of Technology, CHINA

## Abstract

Automatic pavement disease detection aims to address the inefficiency in practical detection. However, traditional methods heavily rely on low-level image analysis, handcrafted features, and classical classifiers, leading to limited effectiveness and poor generalization in complex scenarios. Although significant progress has been made with deep learning methods, challenges persist in handling high-resolution images and diverse disease types. Therefore, this paper proposes a novel approach based on the lightweight Transformer Patch Labeling Network (LTPLN) to enhance the efficiency of automatic pavement disease detection and overcome the limitations of existing methods. Firstly, the input images undergo histogram equalization preprocessing to enhance image quality. Subsequently, the images are evenly partitioned into small patch blocks, serving as inputs to the enhanced Transformer model. This enhancement strategy involves integrating feature map labels at each layer of the model to reduce computational complexity and enhance model lightweightness. Furthermore, a depthwise separable convolution module is introduced into the Transformer architecture to introduce convolutional bias and reduce the model’s dependence on large amounts of data. Finally, an iterative training process utilizing the label distillation strategy based on expectation maximization is employed to update the labels of patch blocks and roughly locate the positions of pavement diseases under weak supervision. Experimental results demonstrate that compared to the baseline model, the proposed enhanced model achieves a reduction of 2.5G Flops computational complexity and a 16% speed improvement on a private pavement disease dataset, with only a 1.2 percentage point decrease in AUC accuracy. Moreover, compared to other mainstream image classification models, this model exhibits more balanced performance on a public dataset, with improved accuracy and speed that better align with the practical requirements of pavement inspection. These findings highlight the significant performance advantages of the LTPLN model in automatic pavement disease detection tasks, making it more efficiently applicable in real-world scenarios.

## 1. Introduction

With the rapid pace of urbanization, a multitude of issues such as cracks, potholes, and other irregular road conditions have emerged, significantly impacting road service life and posing threats to public safety. Timely and precise automated detection plays a crucial role in facilitating prompt road maintenance and enhancing traffic infrastructure. Traditional methods for road disease detection typically rely on traditional image processing [1], manually crafted features [2], and classical classifiers [3]. While these methods operate on relatively straightforward principles, they struggle with defining and extracting features related to diverse diseases, leading to poor generalization. In recent years, propelled by the swift advancements in image processing technology and deep learning theory within the domain of computer vision, deep learning-based road disease detection methods have exhibited notable advantages [4], gradually supplanting traditional approaches. Given the intricacies of road environments, the swift and accurate identification of abnormal pavement conditions has become an urgent and pivotal challenge in this sector.

Current methods for pavement disease detection primarily target specific pavement issues, notably cracks, loose areas, and potholes [5,6], with a significant portion of these studies focusing on pavement crack segmentation [7,8]. In contrast, our study places greater emphasis on identifying whether roads exhibit any form of disease and achieving swift detection of road images with anomalous conditions. The scope of road surface diseases we aim to detect in this paper extends beyond cracks, scattered areas, and potholes to encompass a wider range of ailments, such as repairs, rutting, and even deficiencies in traffic and safety infrastructure along roadways. We term this study automated road disease detection, which can be seen as a generalization of routine road inspection tasks. This task serves as a crucial preliminary step for pavement disease segmentation and represents the core process for pinpointing pavement diseases. Despite being categorized as a binary classification problem for road images, this task poses significant challenges. These challenges stem partly from the uneven illumination, color discrepancies, and complex backgrounds present in road imagery. Furthermore, the diverse array of road diseases, coupled with the potential absence of road facilities, adds to the complexity of the task.

Image classification methods based on deep learning primarily encompass convolutional neural networks (CNNs) such as ResNet [9], DenseNet [10], and EfficientNet [11]. However, these CNN models typically resize images to fixed low resolutions before conducting classification based on the entire image. This resizing process results in the loss of significant image information, particularly for high-resolution images. For instance, ResNet’s input is fixed at 224×224, whereas our road images generally exceed 1200×900 in resolution. The large image size hampers detection model efficiency and makes it challenging to meet real-time requirements for automated road disease detection. Moreover, diseased areas often constitute only a small portion of the entire image. Global-based CNN methods may be susceptible to noise and background variations; for instance, the widely used YOLO series methods [12,13] can lose substantial image information, leading to disease leakage issues. The YOLO method relies on sampling the partition grid of the entire image, and due to the limited receptive field of the convolutional kernel, target features depend solely on local convolution of high quality. Therefore, this paper introduces a novel lightweight Transformer-based patch labeling network (LTPLN) to address automated pavement damage detection. The LTPLN model captures relationships between all pixels through its unique global operation, harnessing contextual image characteristics and compensating for CNN’s local operation limitations. Its lightweight model structure effectively fulfills real-time detection requirements.

In LTPLN, pavement images undergo segmentation into multiple 16-patch blocks. Subsequently, the lightweight Swin transformer serves as the model’s backbone network, responsible for inferring the labels of these patch blocks. The final road image detection results are obtained by performing maximum pooling on the inferred patch block labels. However, this method is limited by the use of only image-level labels. To address this limitation, we draw inspiration from the Expectation-Maximization Patch Label Distillation (EMPLD) strategy [14], which is based on the EM algorithm. This strategy iteratively updates LTPLN solely based on image-level labels. Unlike detection mechanisms based on convolutional CNNs, LTPLN can not only assess detection results at the image level but also roughly pinpoint disease locations within road images through EMPLD in a weakly supervised manner.

The main contributions can be summarized as follows:

1. To the best of our knowledge, we are the first to investigate the task of automated detection of road diseases, which extends beyond specific diseases like cracks, loose areas, and potholes.
2. We have introduced a comprehensive road disease detection dataset, collected from real-world scenarios, covering a wide range of road disease issues. Existing road disease datasets typically include around four disease types, whereas ours comprises over 8,000 high-resolution road surface images, encompassing a greater variety of diseases.
3. We propose a novel Transformer-based automated road surface damage detection method, named the lightweight Transformer-based patch labeling network (LTPLN). This method not only leverages image information efficiently for rapid detection of road diseases but also provides rough localization of the diseases based solely on image labels.
4. We conducted extensive experiments, systematically and empirically comparing the latest state-of-the-art CNN methods in automatic road disease detection. Our work demonstrates a more balanced performance in terms of recognition accuracy and efficiency, better meeting the practical requirements of road detection tasks.

## 2. Related work

### 2.1 Traditional methods

Traditional pavement disease detection methods primarily rely on low-level image analysis, handcrafted features, and classic classifiers. For instance, Shi et al. [15] proposed the CrackForest method, which combines random structured forests and integral channel features for automatic road crack detection. Another method uses a filter bank composed of multi-directional Gabor filters to detect road cracks [16]. Pan et al. [17] utilized images acquired by Unmanned Aerial Vehicles (UAVs) and employed KNN, SVM, random forests, and neural networks to identify pavement cracks and potholes. Hajidemetriou et al. [18] employed traditional Support Vector Machines (SVM) to detect pavement patches. Nhat-Duc Hoang [1] used texture feature extraction and stochastic gradient descent logistic regression for the automatic detection of loose asphalt pavements. Although these traditional methods are relatively simple, they face significant limitations in defining and extracting diverse disease features, leading to poor generalization capabilities and limited effectiveness in complex scenarios.

### 2.2 Deep learning-based methods

With the remarkable success of deep learning in various applications, more researchers have applied advanced deep learning methods to pavement disease detection. Zhang et al. [19] used Convolutional Neural Networks (CNN) to detect crack points for pavement crack segmentation. A VGG-16 DCNN pre-trained on ImageNet was used to classify pavement images as "crack" or "no crack". Xia [20] employed the Single Shot MultiBox Detector (SSD) [21] network to localize pavement diseases. Other researchers used well-known object detection frameworks such as YOLO v2, Faster RCNN, and RetinaNet to locate pavement diseases [22–25]. Fan et al. [26] developed a novel automatic pavement crack detection system that uses CNNs to determine if pavement images contain cracks, followed by adaptive thresholding methods to segment the cracks. These deep learning-based methods exhibit significant advantages over traditional methods, demonstrating higher accuracy and efficiency. However, they still face challenges in handling high-resolution images and diverse types of diseases.

### 2.3 Object detection and image classification

Object detection is a fundamental and challenging problem in computer vision that has garnered extensive attention over the past few decades. Traditional detectors typically use sliding window methods to collect object proposals, then represent them with handcrafted features such as Haar wavelets [27], Histograms of Oriented Gradients (HOG) [28], and Local Binary Patterns (LBP) [29,30]. Learning-based representations like Fisher Vectors (FV) have also been popular in object detection tasks [31]. In recent years, deep learning methods have become mainstream in object detection, leveraging deep convolutional networks to learn robust high-level feature representations. Deep learning-based object detection methods can be divided into two categories: two-stage detectors and one-stage detectors. Two-stage detectors include RCNN [32], SPPNet [33], Fast RCNN [34], Faster RCNN [35], and Feature Pyramid Networks [36]. One-stage detectors jointly optimize object proposal selection and classification, with YOLO [37], SSD [38], and RetinaNet [39] being representative examples. Despite their success in general object detection, these methods face challenges when applied to pavement disease detection due to the need to handle high-resolution images and meet real-time detection requirements.

### 2.4 Challenges in pavement disease detection

Pavement disease detection tasks can be divided into three categories: pavement crack segmentation, pavement crack localization, and specific pavement damage detection. However, comprehensive pavement disease detection (not limited to specific types of diseases) has yet to be systematically studied. Existing datasets such as the Crack Forest Dataset (CFD) [40], CrackTree200 [41], and Crack500 [42] primarily focus on pavement crack segmentation and only contain disease images, limiting their applicability in comprehensive pavement disease detection research. In this study, we introduce a novel deep learning method called the Lightweight Transformer Patch Label Network (LTPLN) for the automatic detection of various pavement diseases. Our collected comprehensive pavement disease detection dataset comes from real-world scenarios, covering a broader range of pavement disease problems, including over 8,000 high-resolution pavement images, providing more extensive and diverse data compared to existing datasets.

### 2.5 Lightweight Transformer Patch Label Network (LTPLN)

To address the limitations of traditional and deep learning-based methods, we propose the LTPLN method. Unlike CNNs, which typically resize images to a fixed low resolution, LTPLN processes high-resolution images by dividing them into multiple 16-patch blocks. A lightweight Swin Transformer serves as the backbone network, inferring the labels of these patch blocks. The inferred patch block labels are then max-pooled to obtain the final detection results. To enhance detection performance, we adopt the Patch Label Distillation (EMPLD) strategy based on the Expectation-Maximization algorithm, which iteratively updates the LTPLN based on image-level labels. This approach allows LTPLN to evaluate detection results at the image level and coarsely localize disease locations in pavement images through weak supervision. Our experimental results show that LTPLN demonstrates a better balance between accuracy and efficiency compared to state-of-the-art CNN methods, making it more suitable for practical pavement detection tasks.

## 3. Lightweight transformer

The lightweight Transformer network has become a popular technique in practical applications because it combines the advantages of the Transformer model with higher efficiency and lower computational costs. This is mainly attributed to several improvements:

1. Reduced Parameters:The lightweight Transformer network achieves lightweightness by reducing the number of parameters in the model. This is often done by reducing the number of hidden units per layer or the number of layers. Fewer parameters mean less computation and memory consumption, making the model more suitable for running in resource-constrained environments.
2. Simplified Structure:Lightweight Transformer networks typically adopt simplified structural designs, such as reducing the number of attention heads or using simpler attention mechanisms. This simplification reduces the model’s complexity and computational requirements while maintaining performance.
3. Feature Reuse:Lightweight Transformer networks may also introduce feature reuse mechanisms by sharing computation results between different layers, reducing redundant computations. This effectively utilizes computational resources and improves model efficiency.

In summary, the effectiveness of lightweight Transformer networks lies in their ability to maintain the advantages of the Transformer model while achieving higher efficiency and lower computational costs through techniques such as parameter reduction, structural simplification, and feature reuse. This makes them suitable for various resource-constrained applications.

### 3.1 Swin transformer

**Fig 1:**
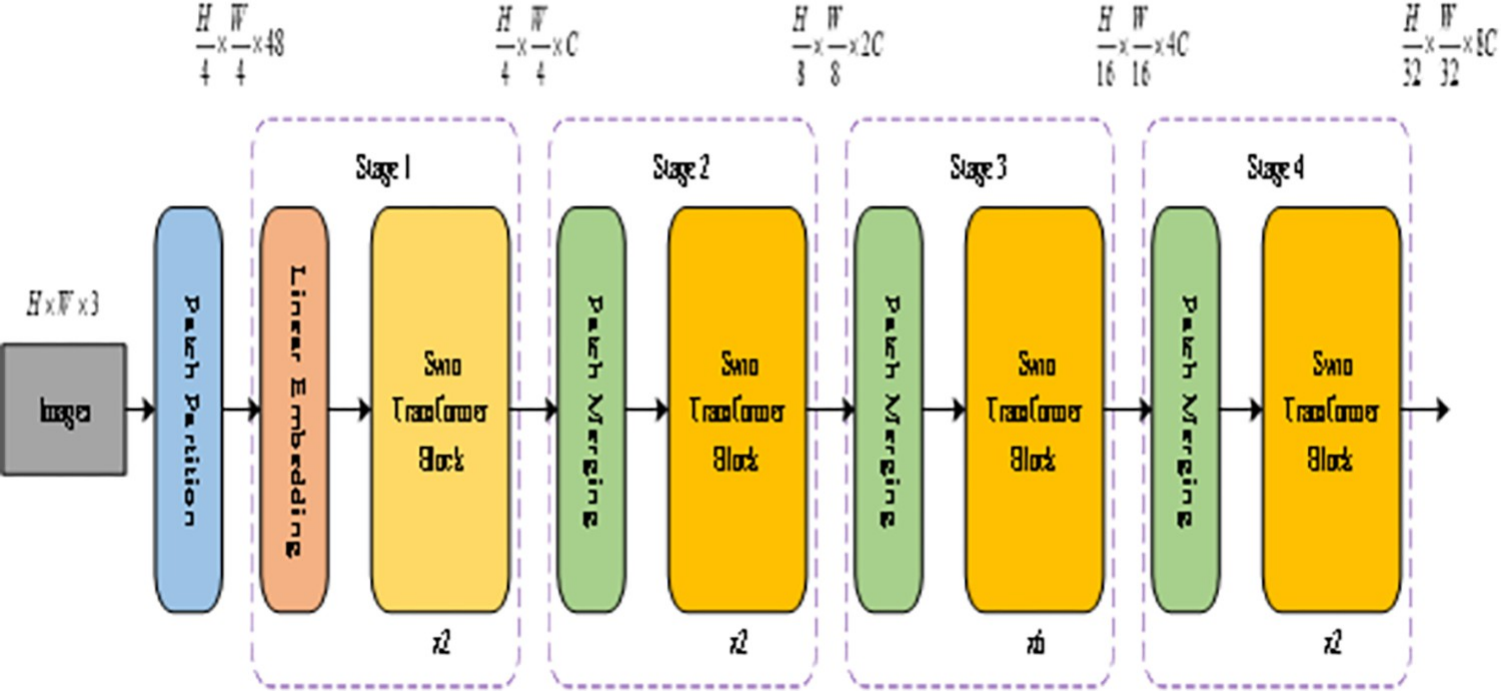
Architecture of Swin-T model.

The Swin Transformer (Swin-T) algorithm [43], introduced by Microsoft Research at the ICCV conference in 2021, employs a hierarchical construction method akin to convolutional neural networks (CNNs) while leveraging the Vision Transformer model [44] to attain multi-scale detection capabilities. This model incorporates the Windows Multi-Head Self-Attention (W-MSA) mechanism for long self-attention to reduce computations and utilizes the Shifted Windows Multi-Head Self-Attention (SW-MSA) mechanism with a mobile window for self-focused multi-head operations. Notably, the SW-MSA mechanism addresses information isolation between Windows, a challenge encountered with W-MSA. The Swin Transformer Tiny model, characterized by minimal parameters, is depicted in Fig 1. Using the Swin Transformer model for pavement disease image classification is illustrated in Fig 1. The network structure of Swin Transformer is composed of four stages, with each stage containing several Swin Transformer Blocks. The Swin-base model used in this paper contains 2, 2, 18, and 2 Swin Transformer Blocks in the four stages, respectively. For the input pavement disease images, the Swin Transformer uses a 4x4 window in the image segmentation layer to segment the images, and the segmented window images are flattened in the channel direction. The height and width of the original image are reduced to 1/4 of the original size, and the number of channels becomes 16 times the original. In the first stage, the image patches are converted into one-dimensional vectors through linear mapping and input into the Swin Transformer Block. In each subsequent stage, the image patches are downsampled through the image patch merging layer. The final output passes through a global pooling layer and a fully connected layer to obtain the classification result of the disease.

**Fig 2:**
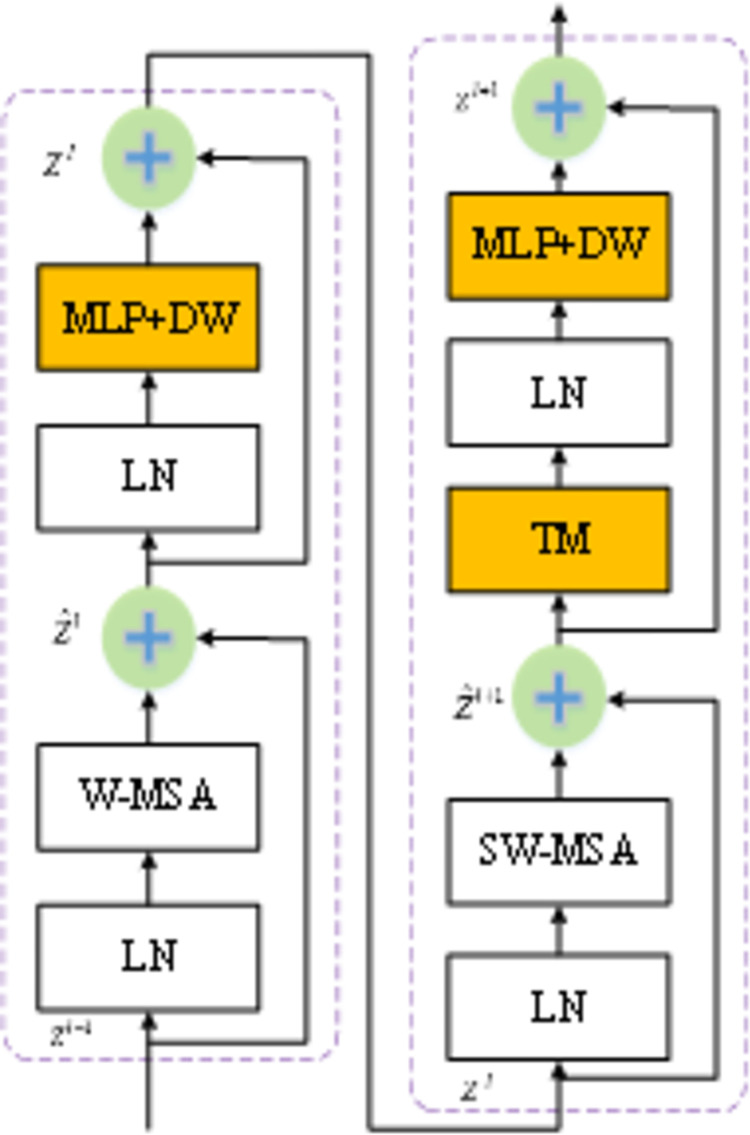
Architecture of Swin Transformer Block.

The core workflow of the Swin-T model comprises three main parts, illustrated in Fig 2. The structure of the two Swin Transformer Blocks is shown in Fig 2. Swin Transformer Blocks always appear in pairs. This is because, in the first block, the Swin Transformer replaces the multi-head self-attention (MSA) module of the original Transformer with the window multi-head self-attention (W-MSA) module. W-MSA does not compute the attention between all pixels in the entire image; instead, it divides the image into several windows and computes the attention between pixels within each window, without interacting with other windows.

Here’s the refined version of the description:

1. The input image undergoes segmentation in the block segmentation layer, where adjacent 4x4 pixels form individual blocks. The resulting feature vectors are flattened along the channel dimension and then linearly transformed via the linear embedding layer.
2. The model employs four stages to generate feature maps of varying sizes. The last three stages utilize block fusion for downsampling, followed by repeated stacking of Swin Transformer Block modules. Each Block module integrates both W-MSA and SW-MSA structures, typically used in pairs.
3. The final connection consists of standard normalization, global pooling, and fully connected layers for image classification tasks.

The Swin Transformer has demonstrated strong performance across various computer vision tasks such as image classification, object detection, and image segmentation. However, its computational demands are considerable, necessitating further lightweight model improvements for effective deployment in road automation detection applications.

### 3.2 Modified transformer

Given the demanding requirements for high-speed and precise automated pavement disease detection, we have implemented lightweight enhancements to Swin-T while upholding its accuracy. These specific improvements, depicted in orange in Fig 2, are detailed as follows:

1. Integration of the Token Fusion Module (TM) [45]: This lightweight model outcome, TM, has been strategically inserted between the SW-MSA and the multilayer perceptron within each Swin-T Block. This integration significantly reduces the computational burden of the model.
2. Incorporation of the Depthwise Separable Convolution Module (DW) [46]: Recognizing the challenge of Transformer models relying heavily on extensive training data, we have introduced the DW module into the multi-layer perceptron. This inclusion enables the model to effectively adapt to smaller datasets.

The resultant lightweight model, enhanced with TM and DW modules, is denoted as MSTTM (Modified Swin Transformer Tiny Model).

#### 3.2.1 Token merging

After segmenting the image into multiple small patch blocks and converting them into marker vectors, the Transformer self-attention mechanism computes the relationships among each marker vector and all others. This results in a model complexity proportional to the square of the input marker vectors, necessitated by the Transformer architecture for processing high-resolution images. To mitigate the computational burden, a commonly adopted method is to prune the marker vectors, albeit at the cost of reduced computational accuracy. However, this pruning strategy presents several drawbacks. Firstly, it entails introducing supplementary neural networks to compute scores for each marker vector and decide which ones to retain. Secondly, pruning may lead to the loss of critical information, necessitating careful determination of the appropriate pruning ratio.Given these limitations, the introduction of Token Fusion Modules without additional training emerges as an effective lightweight alternative within the Swin Transformer model framework, particularly when compared to marker vector pruning. This approach not only reduces computational complexity but also sidesteps the challenges associated with information loss and the need for additional neural network components.

**Fig 3:**
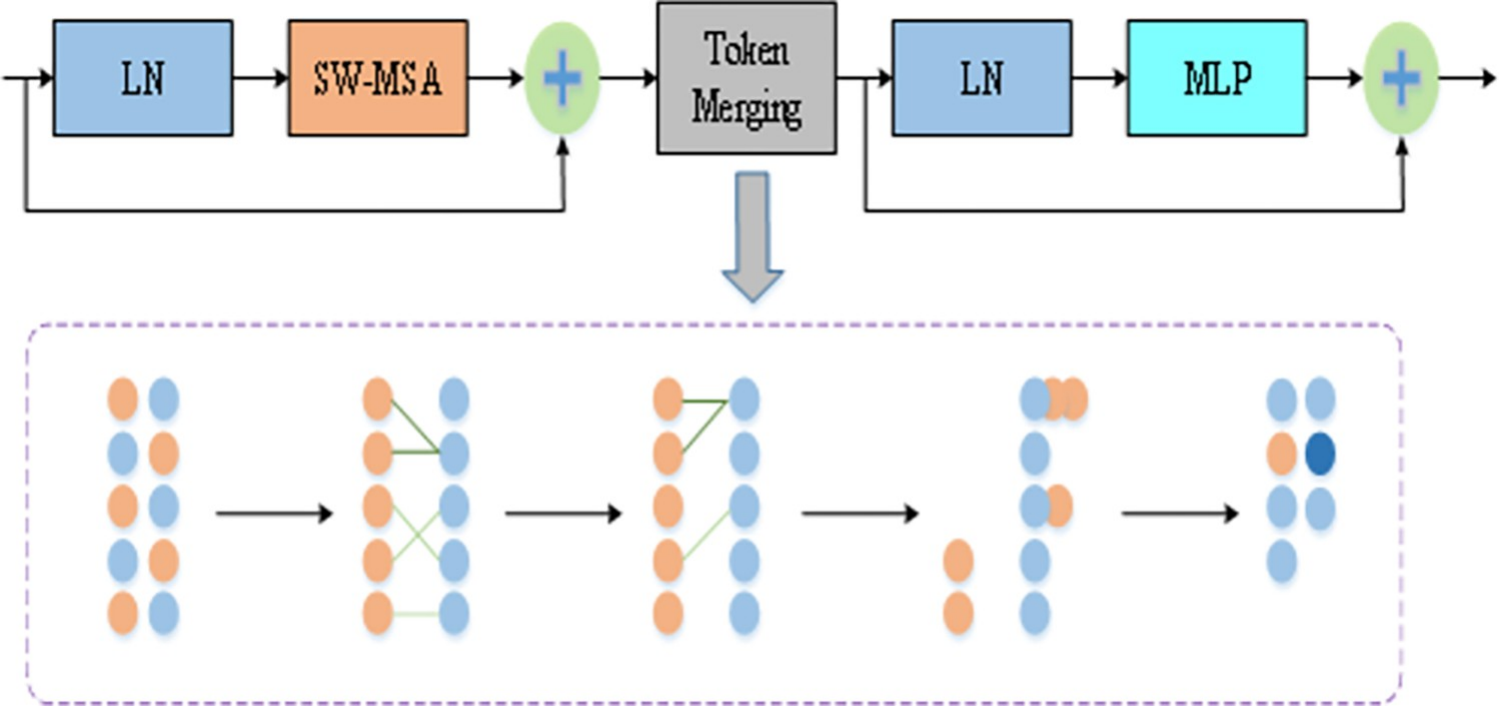
Token merging module.

The self-attention mechanism inherently captures the correlation between marker vectors during the transpose and query matrix dot product operations.As shown in Fig 3, Specifically, it calculates attention weights by extracting the query *(Q*), key (*K*), and value (*V*) matrices of each marker vector, thereby gauging the similarity between them. This similarity can be quantified using the cosine distance of the bond matrix mean.

In the Token Fusion Module positioned between the SW-MSA module and the MLP, the self-attention calculations within the SW-MSA module ascertain the similarity between marker vectors, ensuring seamless propagation of marker vectors through the MLP post-fusion. A binary soft matching algorithm within a specific Token Fusion Module swiftly identifies and matches similar marker *r* vectors, thereby accurately reducing redundant marker vectors. The process unfolds as follows:

1. Divide the input marker vectors into sets A and B within the module.
2. For each marker vector in set A, find its most similar counterpart in set B and establish connections.
3. Retain the top *r* most similar connections.
4. Merge the still-connected marker vectors with the average value of each item.
5. Output the amalgamated set as the final result.

**Fig 4:**
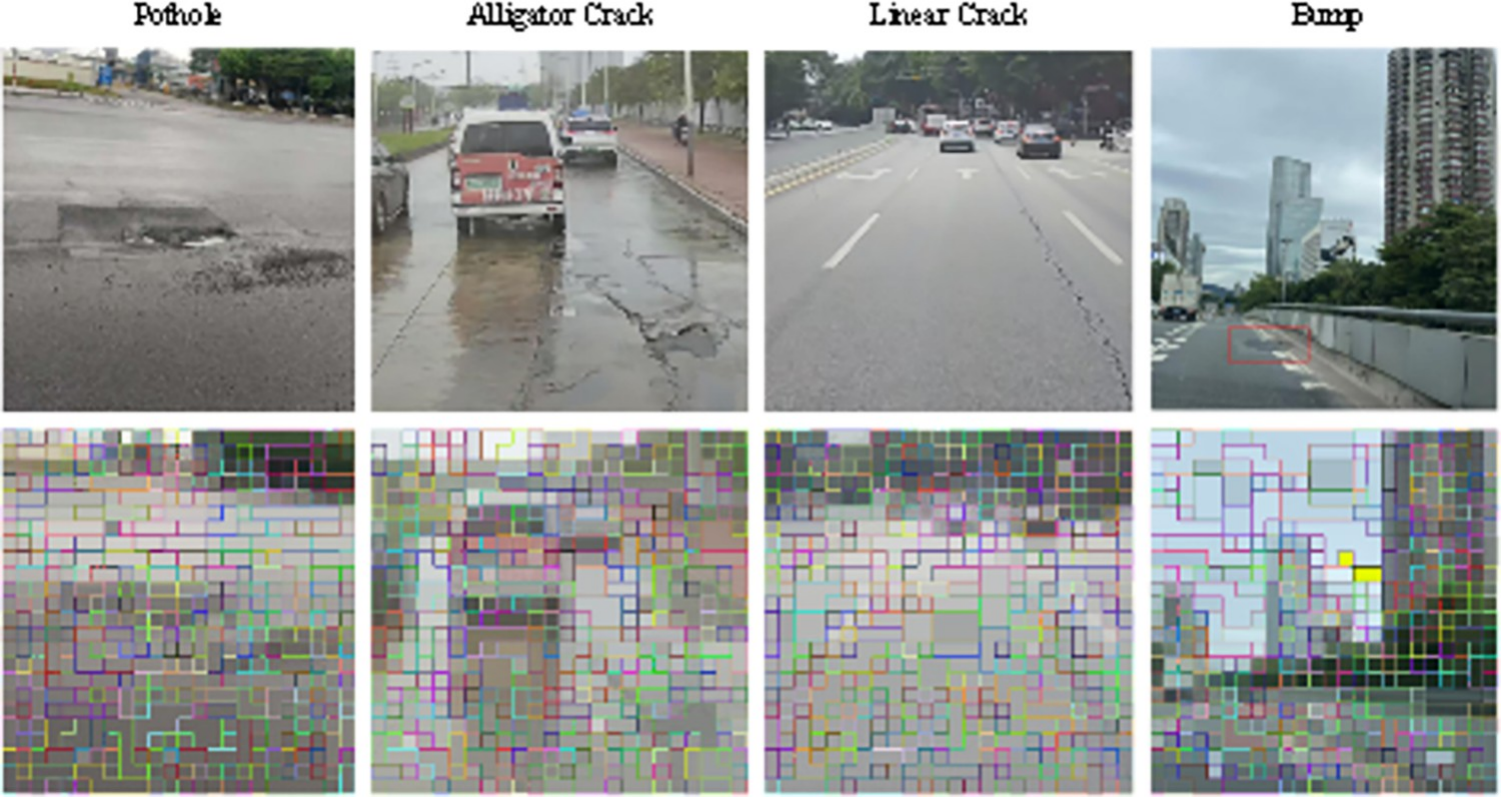
Token merging for visualization. Republished from Fig 4 under a CC BY 4.0 license, with permission from Feng LIU, original copyright 2024.

The reduction in feature map output at each stage after Token Fusion effectively reduces computational load in subsequent stages, contributing to the model’s lightweight design. Additionally, to illustrate the impact of Token Fusion further, a visual analysis of the fusion process was conducted, as depicted in Fig 4. During fusion, marker vectors corresponding to the same background block merge, while those from different background blocks remain distinct. This approach ensures that fused marker vectors do not introduce excessive interference in disease target identification.

#### 3.2.2 Depthwise separable convolution

The challenges faced by Transformer models in image classification tasks, such as their dependency on large training datasets compared to CNN models, are well-understood. One fundamental reason for this requirement is the absence of CNN-like convolution pooling in Transformers, which aids CNNs in achieving robust detection even with smaller datasets. While Transformers excel at capturing global context through their self-attention mechanism, they may lack prior knowledge, necessitating extensive data for learning.To enhance classification accuracy and introduce convolution properties into the Swin-T model, we have introduced convolution operations, specifically deeply separable convolution. This addition aims to strike a balance between improving model performance and meeting the lightweight requirements essential for efficient processing.

**Fig 5:**
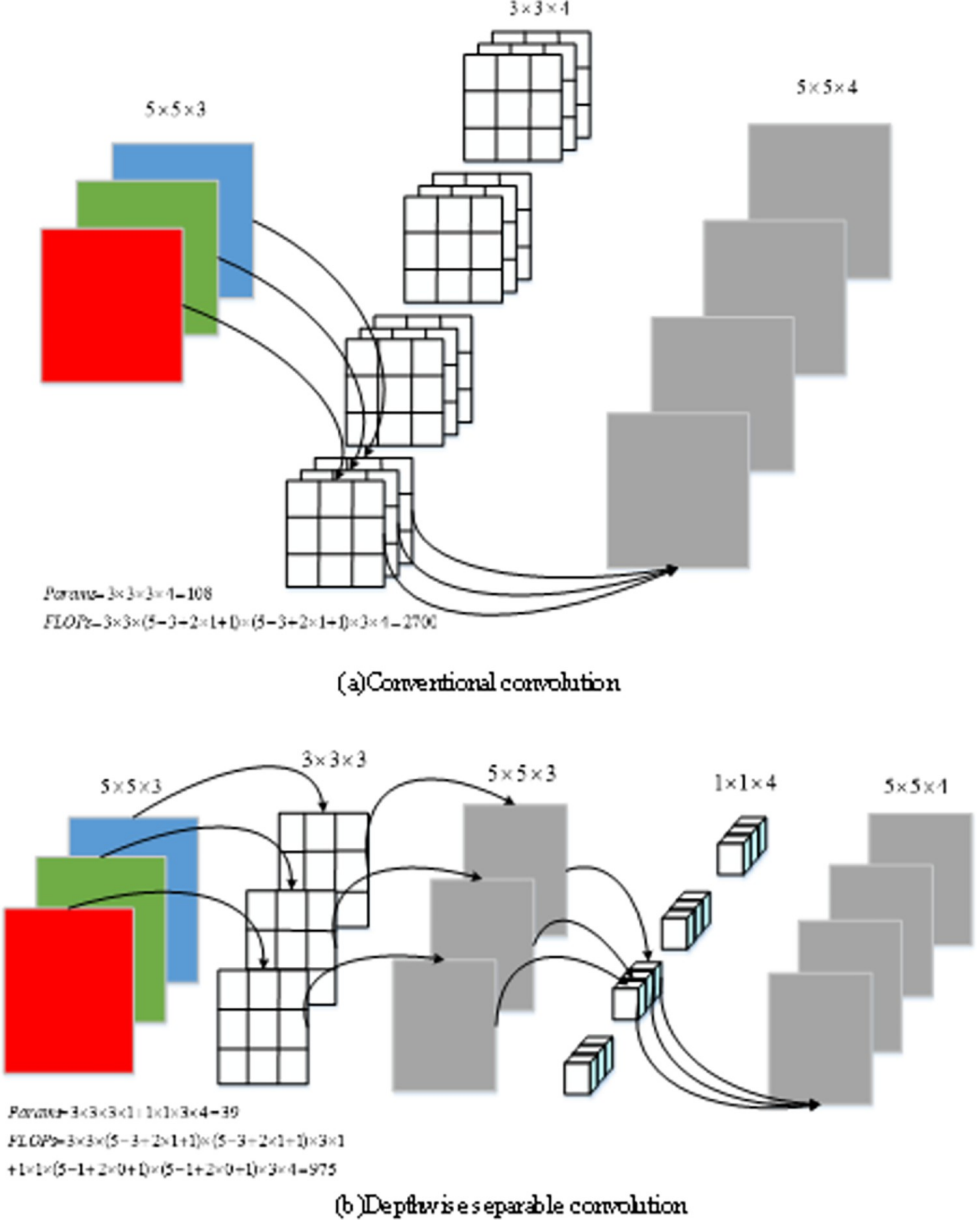
Conventional convolution and depthwise separable convolution.

Depth separable convolution mainly consists of point-by-channel volume and channel-by-channel convolution. Specifically, a channel-by-channel convolution is only responsible for the information processing of one channel to generating a new feature graph, which is weighted based on the positions of the global input feature graph in the channel direction. Compared with the conventional convolution, the depth of separable convolution has a large reduction in the number of parameters and calculation, which is more in line with the requirements of lightweight. As shown in Fig 5, the number of parameters (Params) of conventional convolution is 108, and the calculated quantity (FLOPs) is 2700; the number of point convolution is 12 and the calculated amount is 300; the number of parameters for channel convolution is 27, and the calculated quantity is 675. However, compared with the number of parameters of conventional convolution 108 and 2700, the overall number of deeply separable convolution is 27 + 12 = 39 and the overall computation is 675 + 300 = 975, decreasing about 2/3. It can be seen that the deeply separable convolution can better meet the lightweight requirements for the same inputs and outputs.

In the process of using deeply separable convolution, the input data of the Swin-T model MLP module is first adjusted to a two-dimensional feature schema, and then goes through 11 convolution, GELU activation function and Dropout operations. Then the depth separable convolution is added, and after 11 convolution and Dropout operation, the final output is adjusted to one-dimensional sequence format. Such a design helps the model to introduce inductive bias and improve the model performance under lightweight requirements.

## 4. Transformer patch label network

### 4.1 Method overview

**Fig 6:**
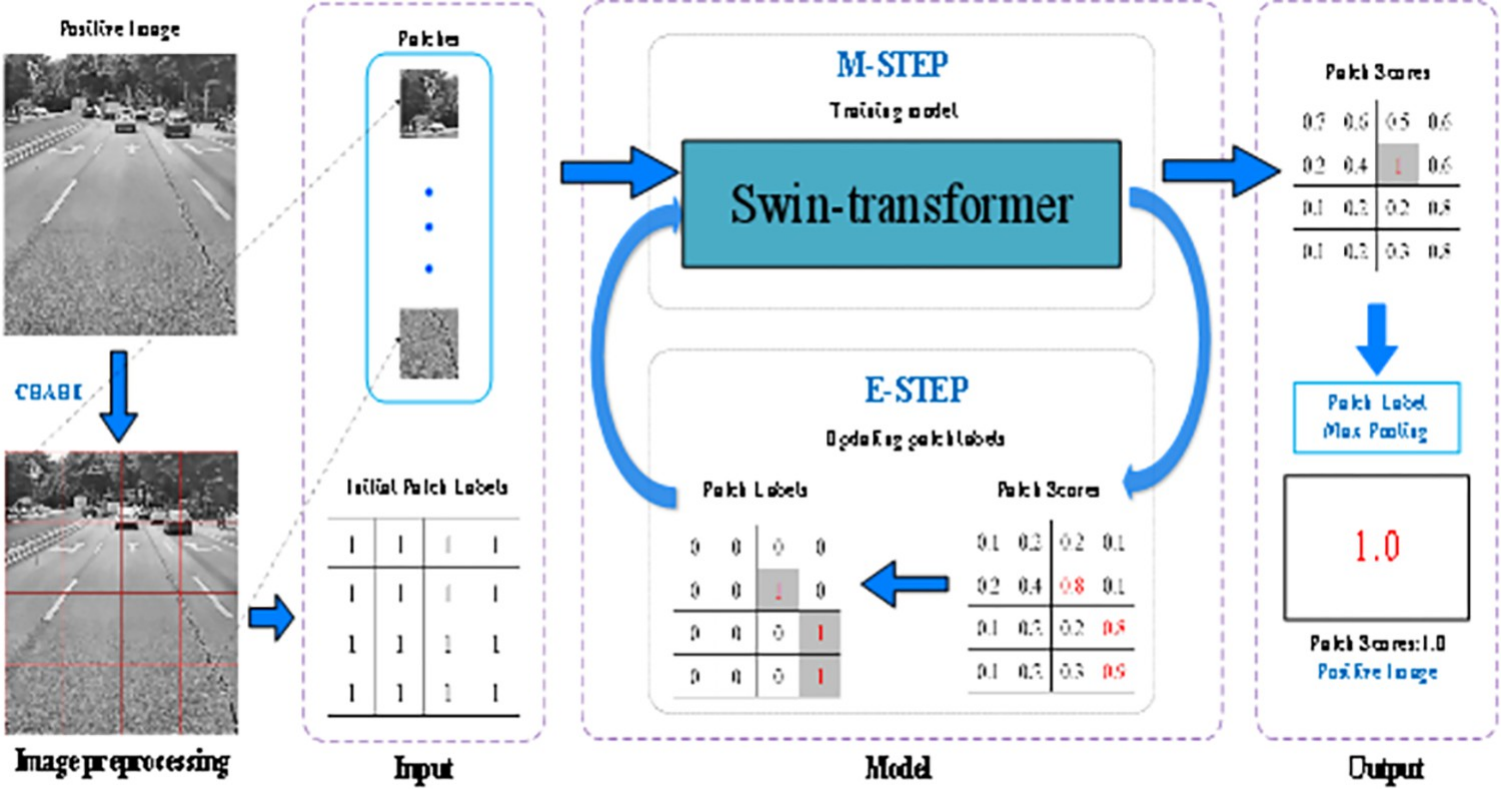
LTPLN model structure diagram. Republished from Fig 6 under a CC BY 4.0 license, with permission from Feng LIU, original copyright 2024.

The methodology behind this approach draws inspiration from recent breakthroughs in Transformer research for computer vision [43–46]. By amalgamating advanced computer image processing techniques with machine learning algorithms, our model design aims to achieve continuous improvements in performance. In our Token fusion patch labeling network (TPLN), the initial step involves preprocessing road images using contrast-limited adaptive histogram equalization (CLAHE) to mitigate adverse effects stemming from uneven lighting conditions. Subsequently, the processed images are segmented into patches, and LTPLN endeavors to predict patch labels. Finally, pavement image labels are derived through maximum pooling of their patch labels. Central to our methodology is LTPLN. However, training LTPLN directly poses challenges due to the absence of patch labels during training; only image labels are available. To circumvent this limitation, we propose an EM patch block label distillation strategy to iteratively optimize LTPLN, leveraging reasonably initialized patch labels. The model structure is illustrated in Fig 6.

### 4.2 Preprocessing and input

Because the pavement images were taken at different times and in different areas, the images had severe uneven illumination. To suppress the negative effects of illumination, the pavement images were processed with CLAHE. Empirical analysis shows that this pretreatment can improve the detection performance. Traditional convolutional neural networks (Convolutional Neural Networks, CNN), such as Faster RCNN, YOLOv3 and YOLOv7, usually require smaller input image sizes, such as 460×460 and 320×320, while the road images on our dataset are taken from on-board cameras and mobile phones, and the image sizes are large. The method in this paper is to adjust the high-resolution images to 384×384 size images, and then divide the images into patches and realize detection by using the LTPLN model as the backbone network. In this way, the image global information can be fully utilized, and patch labels can be generated. In this paper, simply following the non-overlapping image block strategy and fixing the patch size to 96×96, the size size of the backbone network input for feature extraction was set to 384×384. Suppose that each image is divided into m 96*96 blocks. Such steps can be expressed mathematically as follows:

$$x_{i}=H(I_{i})≔\{p_{1}^{i},\cdots ,p_{t}^{i},\cdots p_{m}^{i}\}$$ (1)

where *x*_*i*_ is the i-th preprocessed image, *H(I*_*i*_*)* is the CLAHE operation, and $p_{t}^{i}$ represents the t-th patch of the image. And m is the number of patches, m is 4×4 = 16.

### 4.3 Model

In this paper, the lightweight LTPLN network optimized by Token fusion (TM) and deep separable network (DW) is selected as the label for inferred patch of the backbone network. The model is represented as follows:

$$g_{t}^{i}=M_{\alpha }(p_{t}^{i})$$ (2)

where *M*(•) is the mapping function of the model, and *α* is its associated network parameter. Is the predicted value of the true patch block label $g_{t}^{i}\in (0,1)$ equal to 1 when a disease is present and equal to 0 when there is no disease. Because only the whole image tag *y*_*i*_ is available, and the patch block tag $l_{t}^{i}$ has no real tag available, this hinders the normal training of LTPLN. In this section, the LTPLN is trained by using an iterative method called a label distillation strategy based on the EM algorithm. The basic idea is to provide a reasonable patch label initialization for training LTPLN and to retrain LTPLN against the new labels inferred by the previous version of LTPLN. These steps were performed iteratively until convergence. Considering the training step M and the label reasoning step E, the iteration scheme is very similar to the idea of the expectation maximization (EM) algorithm, and the patch labels will be gradually refined during the iteration because patches from normal pavement images are always normal, so these reliably labeled data and diseased pavement images enable continuous iterative optimization of LTPLN.

1. Initialization of the patch label ${\tilde{l}}_{t(0)}^{i}=y_{i}\subset \{0,1\}$ treats the image label as the initial label of its patch. In this case, patch labels from normal pavement images are credible, while patch labels from diseased pavement images are suspicious because the diseased areas generally do not cover all areas of the image.
2. Maximization (M) Step: To obtain the network parameter of LTPLN *α*_*j*_ in the j th iteration.
3. Expectation (E) Step: Step E is to use the trained LTPLN to infer the label of the patch. According to Eq 2, each patch can obtain a label prediction value, called the confidence equal number $g_{t(j)}^{i}$. Then, an image-based level-aware threshold (Image Rank Threshold, IRT) protocol is used to adaptively update the labels of each patch based on the confidence score. Only the patch label for the diseased road image is updated here, because the patch label for the normal image should always be 0 ("normal"). The IRT is the core of the E-step.

Image-based level perception threshold (Image Rank Threshold, IRT): patch *p*_*t*_ in disease image *x*_*i*_ is updated by IRT: disease patch should meet the condition: the ratio patch with confidence score higher than the number of diseases to the total number *s*_*j−1*_ 1 in the previous iteration, and the initialization can be automatically calculated in each iteration,*s*_*0*_ = 0.5;

Such a label updating strategy can be mathematically denoted as follows:

$${\tilde{l}}_{t(j)}^{t}=\{\begin{array}{cc} 1, & g_{t(j-1)}^{i}<\min(s_{j-1},\tau_{i}(r))and\;y_{i}=1\\ 0, & others \end{array}$$ (3)

4. Prior knowledge is biased toward cross-entropy (Prior Knowledge Biased Cross-Entropy, PKCB): We believe that the labels of lesion patches produced by LTPLN in the last iteration are more reliable than lesion blocks with lower scores, and that the improved LTPLN should also suppress normal patches with high confidence scores. Therefore, the distribution of confidence scores and the patch labels obtained in the last iteration was considered as prior knowledge and combined to design a weighted scheme for cross-entropy. We introduce this new cross-entropy loss called prior knowledge bias Prior Knowledge Biased-Entropy (PKBCE) into LTPLN.

$$\begin{array}{c} L_{j}=-\frac{1}{nm}\sum_{i=1}^{n}\sum_{t=1}^{m}\frac{g_{t(j-1)}^{i}}{s_{j-1}}\{{\tilde{l}}_{t(j)}^{i}\log(g_{t(j)}^{i})\\ +(1-{\tilde{l}}_{t(j)}^{i})\log(1-g_{t(j)}^{i}) \end{array}$$ (4)

the $\frac{g_{t(j-1)}^{i}}{s_{j-1}}$ is considered as the normalized version of $g_{t(j-1)}^{i}$, and a higher $g_{t(j-1)}^{i}$ implies that the corresponding patch is paid more attention to the next training.

### 4.4 Output

After the optimization of LTPLN converges, the trained LTPLN model is used to label the patches of test images. The detection label of a test image *x*_*i*_ is obtained by applying maximum pooling to its patch labels, $y_{i}=\max(\{{\tilde{l}}_{t}^{i}|\forall t\})$. This strategy ensures that the final detection label inference is independent of the number of patches in an image. Consequently, our model is capable of handling images of any resolution.

Algorithm 1 presents the specific steps of our approach.

Algorithm 1. LTPLN pavement disease detection.

1: [Initialization]

2: ${\tilde{l}}_{t(0)}^{i}=y_{i}\subset \{0,1\}$

3: While $\sum_{i=1}^{n}\sum_{t=1}^{m}\left|{\tilde{l}}_{t(j-1)}^{i}-{\tilde{l}}_{t(j)}^{i}\right|>0$ do

4: [M-step]

5 Training *M*_*α*_(●) with {*x*_*i*_|∀*t*}, $\left\{{\tilde{l}}_{t(j-1)}^{i}|\forall t,i\right\}$ and *α*_*j*−1_ using the losss in Eq 4

6: [E-step]

7: Obtaining the $g_{t(j)}^{i}$ for each patch and updating the labels of all patches based on Eq 3 with *r* and *s*_*j*−1_

8: Updating *s*_*j*_ and the loss function in Eq 4 based on new labels

9: end while

10: The detection label is the maximum pooling of the labels of its patches inferred by the trained LTPLN.

## 5. Experiments and analysis

### 5.1 Data sets and evaluation metrics

#### 5.1.1 Data set

In this experiment, we utilized two datasets: the private training dataset (PTD) and the publicly tested dataset (OTD). The PTD underwent random partitioning into training, validation, and test sets, maintaining a ratio of 7:2:1. Subsequently, the OTD was employed to assess the model’s performance and select the optimal model from the PTD based on its performance on the OTD’s test set.

**Fig 7:**
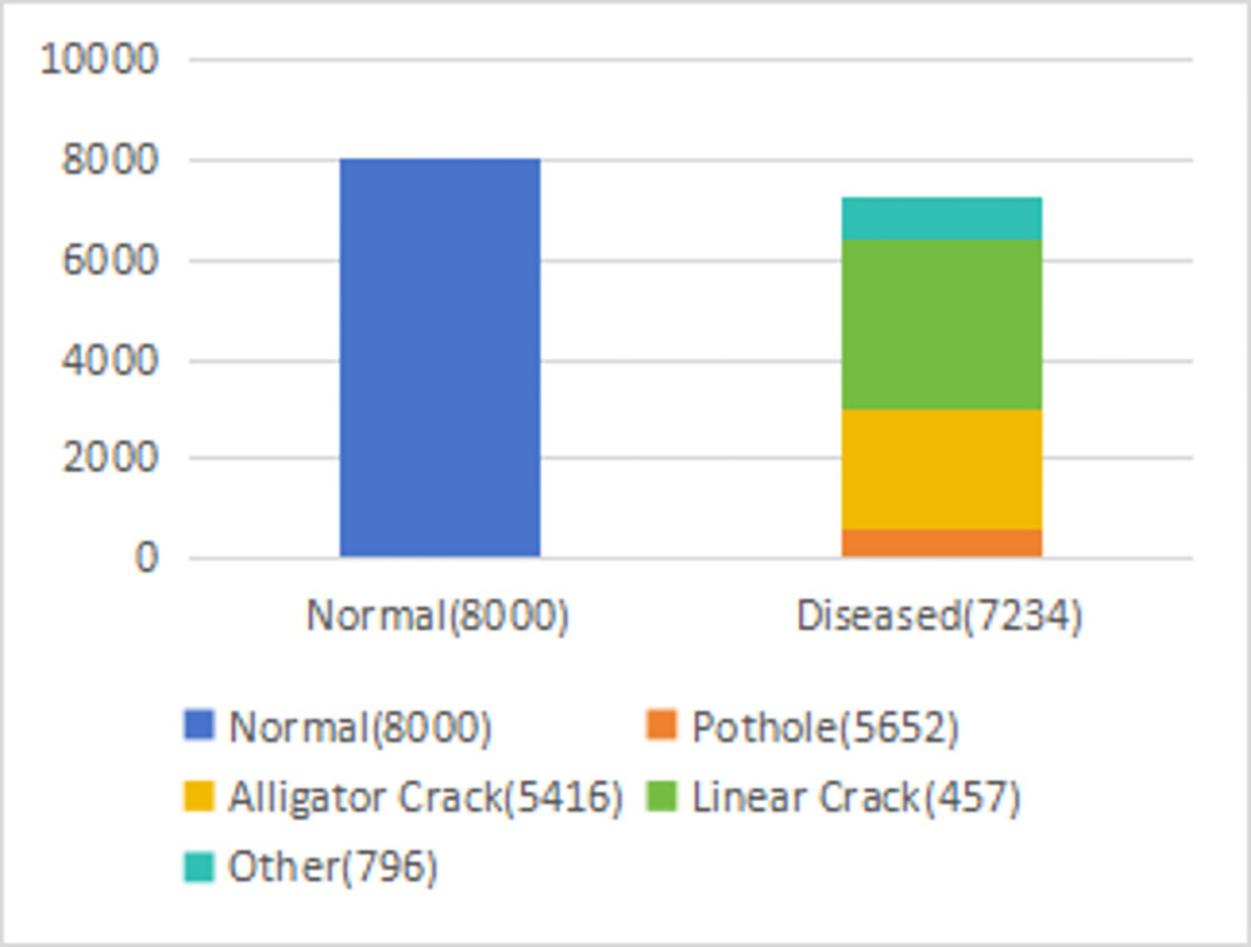
Disease distribution map of the GZDL-BD.

(1) The private dataset PTD comprises the Guangzhou Road Research Institute road pavement disease detection dataset (GZDL-BD), encompassing 80,000 images obtained from on-board video capture and human photography. This dataset encompasses various disease types, including pit, transverse fissure, longitudinal fissure, massive fissure, crack, loose, embrace, and vehicle-related issues. These diseases were broadly categorized into line fissure, mass fissure, pit, and other types. Fig 7 illustrates the distribution of these disease types. To construct the training set, we randomly selected 12,820 diseased pavement images and 12,000 normal pavement images from GZDL-BD, while the remaining dataset was allocated to the test set. The test set comprised 2,129 images of diseased road surfaces and 2,000 normal images. The GZDL-BD dataset is publicly available and can be accessed at https://doi.org/10.5061/dryad.6t1g1jx6w.

(2) The public dataset OTD is a combination of two commonly used pavement disease datasets: the Crack Forest Dataset (CFD) [40] and the Automatic Pavement Disease Detection Dataset (RDD2022) [47]. Initially, the CFD contained 155 images with a resolution of 480×320, while the RDD2022 dataset featured four types of road diseases: longitudinal, transverse, massive (cracked), and pits. Since these datasets focus on road disease detection tasks, all samples are disease images (positive samples), lacking normal images (negative samples). To address this, normal images were synthetically generated by replacing diseased pixels with adjacent normal pixels. Subsequently, low-quality generated normal images were filtered out, retaining only high-quality ones. The CFD dataset then comprised 255 diseased images and 214 recovered normal images, while the RDD2022 dataset contained 800 diseased images and 850 recovered normal images. These integrated images form the Open Test Dataset (OTD) for testing. Notably, the pavement disease detection model discussed in this section was exclusively trained on the GZDL-BD dataset without any fine-tuning on the OTD. This decision was based on the model’s ability to generalize across datasets, including TLIN and other state-of-the-art methods.

#### 5.1.2 Evaluation indicators

The detection of pavement diseases through image classification is fundamentally a binary classification task. To evaluate the performance of our proposed method, we employ three key metrics: precision, recall, and the area under the receiver operating characteristic curve (AUC). These metrics were specifically chosen due to their relevance to the research objectives and their ability to provide a comprehensive assessment of the method’s effectiveness.

Precision measures the accuracy of the positive predictions made by the model. It is defined as the ratio of true positive predictions to the total number of positive predictions (both true and false positives). Mathematically, precision *P* is expressed as:

$$P=\frac{TP}{TP+FP}$$ (5)

where *TP* represents the number of true positives and *FP* represents the number of false positives. Precision is crucial in the context of pavement disease detection because a high precision indicates that the model is effective at identifying diseased samples without mistakenly labeling normal samples as diseased. This reduces the risk of unnecessary maintenance actions based on false positives.

Recall (or sensitivity) measures the model’s ability to correctly identify all true positive cases. It is defined as the ratio of true positive predictions to the total number of actual positive cases (true positives and false negatives). Mathematically, recall *R* is expressed as:

$$R=\frac{TP}{TP+FN}$$ (6)

where *FN* represents the number of false negatives. Recall is particularly important in this research because missing diseased samples (false negatives) can have serious consequences, potentially leading to unsafe road conditions. Therefore, a high recall is essential to ensure that the model detects as many true positive cases as possible.

AUC (Area Under the Curve of the Receiver Operating Characteristic) provides a comprehensive measure of the model’s ability to distinguish between positive and negative classes across different threshold settings. The AUC value ranges from 0 to 1, with higher values indicating better overall performance. AUC is mathematically defined as:

$$AUC=\frac{1}{N_{P}\cdot N_{n}}\sum_{i=1}^{N_{p}}\sum_{j=1}^{N_{n}}\prod (S_{i}>S_{j})$$ (7)

where *S*_*i*_ and *S*_*j*_ are the scores of the positive and negative samples, respectively, *N*_*p*_ and *N*_*n*_ represent the number of positive and negative samples, and Π is the indicator function that equals 1 if *S*_*i*_ > *S*_*j*_ and 0 otherwise. AUC is valuable because it reflects the model’s potential performance independent of any specific threshold, providing a robust indicator of its overall discriminative ability.

These metrics were selected because they align with the primary research objectives of accurately detecting and classifying pavement diseases. Precision and recall directly address the need to minimize false positives and false negatives, respectively, which are critical for practical applications in road maintenance and safety. AUC, on the other hand, offers a holistic view of the model’s performance across various threshold settings, ensuring that the assessment is not biased by a particular threshold choice.

By using these evaluation metrics, we can thoroughly assess the proposed method’s performance, ensuring that it meets the necessary standards for practical implementation in pavement disease detection and classification tasks. This comprehensive evaluation helps to validate the effectiveness of the method and its potential for real-world applications.

### 5.2 Experimental environment

**Fig 8:**
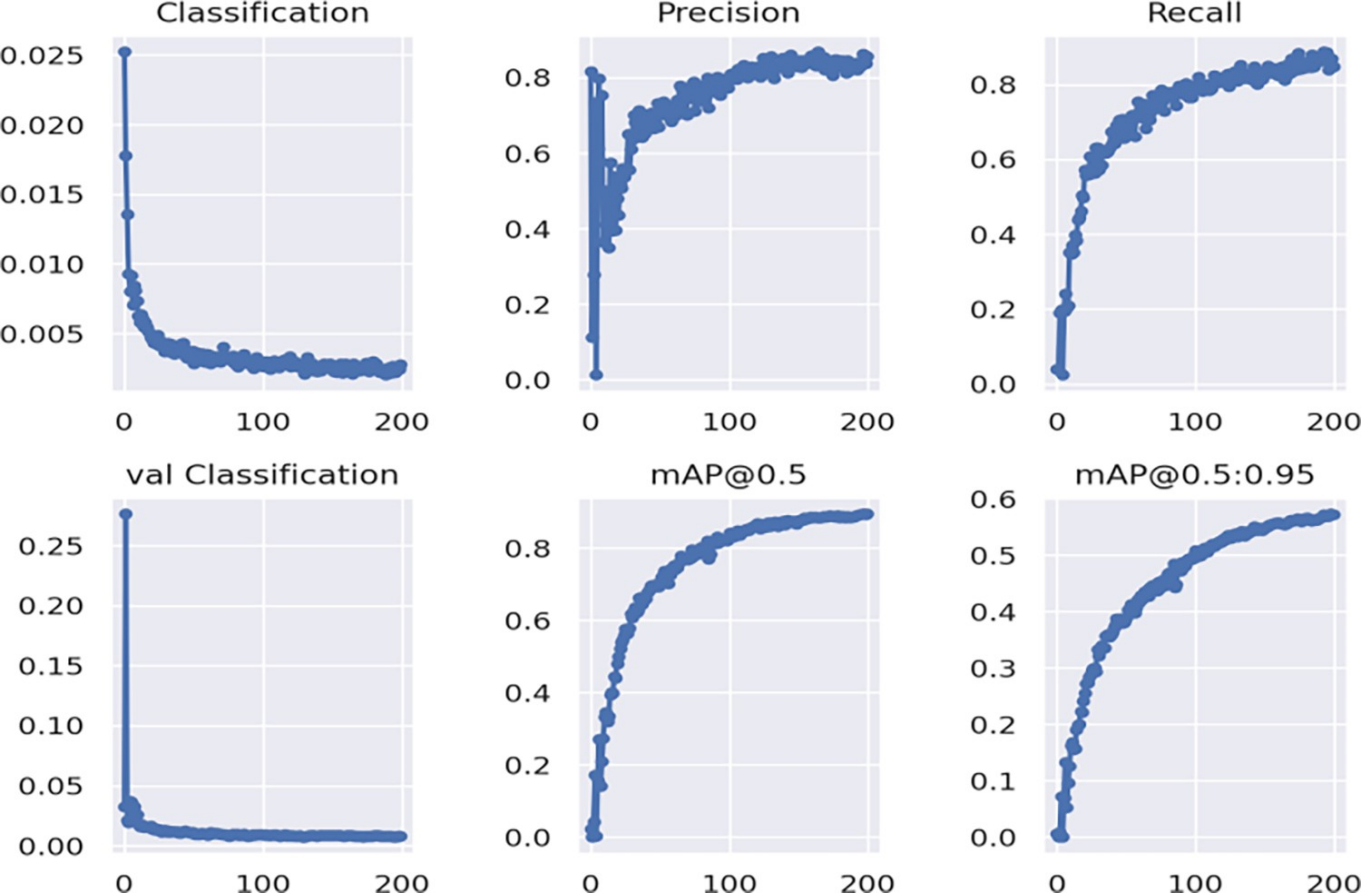
Model the experimental results curve.

The experimental setup utilized an Intel Core i7-9700 processor running at 3.00 GHz with 412 GB of memory, alongside an NVIDIA TITAN GPU for graphics processing. The model was developed using the PyTorch framework within a Python 3.8 programming environment, leveraging CUDA 11.4 parallel architecture for enhanced computational performance. During training, the batch size was set to 16 due to GPU memory constraints, with an input size of 384×384. The training process comprised 200 epochs, utilizing the Adam optimizer with an initial learning rate of 0.001 and dynamic learning rate adjustment. A Dropout value of 0.2 was employed to prevent overfitting. Fig 8 illustrates the model’s loss convergence over training time, demonstrating no signs of overfitting.

### 5.3 Ablation experiments

To evaluate the impact of various improved modules on model performance, we designed five sets of experiments to analyze different improved modules for each set of experiments using the same training parameters. The detection results of the model performance are shown in Table 1, and each module is superimposed in sequence. Comparing the performance of Swin-T and Swin-T + Token fusion (+TM), adding Token fusion provides a 16% improvement in FLOPs and FPS speed metrics by about 24%, indicating that the Token fusion module can accelerate the model to some extent and help reduce the computational amount. Although the AUC and Precision decreased by 3.4% and 0.035 from the baseline model, this is consistent with the expectation of sacrificing partial precision in a lightweight model. The introduction of deeply separable convolution (+DK) caused the system to increase AUC by 1.2% and Precision by 0.013 due to some inductive bias, but the model FLOPs only slightly increased by 1.4%. The data preprocessing (+CLAHE) of the improved model improved the AUC by 0.3% and 0.003 without changing FLOPs and speed, indicating that the image preprocessing operation can improve the recognition accuracy of the model. Finally, the model was trained (+ EM), and the image patch block successfully learned the label information from the whole image. The above improvement strategy finally achieved 14% decrease in FLOPs and 16% improvement in the AUC loss of 1.2% and Precision score decrease of 0.011, demonstrating the effectiveness of the four improvement methods in the balance of speed and accuracy.

**Table 1:** Comparative results of the ablation experiments.

Model	Precision	Recall	AUC/%	PLOPs/G	FPS
Swin-T	0. 935	0. 935	95.3	17. 5	50
+TM	0. 900	0. 901	92.1	14. 7	62
+DK	0. 913	0. 891	93.2	14. 9	60
+CLAHE	0. 916	0. 905	93.5	14. 9	60
+EM	0.924	0.925	94.2	15.0	58

In addition, the resolution of the images taken by the on-board camera is 2560*1440 in the daily inspection task of actual road diseases, the detection time requirement is 15 FPS, and the classification accuracy requirement is greater than 90%. After the deployment of the model in the cloud service platform, the reasonable time of each image is about 15ms, and the image to be tested is divided into 16 small patches of 9696 size into the model (model input size is 384×384), which meets the actual requirements of automatic detection time, and the final detection speed is about 23 fps. At the same time, the classification AUC accuracy of the improved model has reached 93.6%, which is already fully competent in terms of detection accuracy [48].

### 5.4 Comparison of the experiments

In order to evaluate the detection performance of the improved model, we conducted comparative experiments with the mainstream image classification models, including a variety of classic common image classification networks (such as ResNet series [9], DenseNet series [10], EfficientNet series [11] and Vision Transformer series [49]), and the latest achievements in the field of image classification (ConvNext series [50,51] and EVA series [52,53]). The experimental results are shown in Table 2, where the input image dimensions are 384×384. By comparing the table, it can be found that although the convolutional neural network ResNet-34 is better than the improved model in both FLOPs and speed (FPS), its accuracy is 7.2% lower than that of the improved model. However, the DenseNet169 and EffecientNet-v2 models, although having smaller FLOPs, are inferior to the improved models in both accuracy and speed. The MobileViT-v2 model achieved the best performance in detection speed, but there is still a gap in accuracy. Finally, although the improved model decreased by 0.9% and 1.8% compared with the AUC index of ConvNext-T and EVA02 models, the speed increased by about 16% and 480%, which is more in line with the requirements of industrial real-time detection. Therefore, considering the detection accuracy and speed, the improved model has a more balanced performance in the homemade data set than the current mainstream model of image classification.

**Table 2:** Results of comparison experiments.

Model	AUC	PLOPs/G	FPS
Resnet-34	87. 4	14. 6	68
DenseNet169	91. 1	13. 7	32
EffecientNet-v2	90. 6	11. 6	28
MobileViT-v2	90. 5	9. 8	73
ConvNext-T	95. 1	17. 8	50
EVA02	95. 8	87. 5	10
Ours	94.2	15.0	58

Table 3 shows the results of the contrast experiments on the RDD2022 dataset. According to the data in Table 3, our method showed relatively outstanding performance on the RDD2022 dataset, achieving an AUC of 90.10%, which is comparable to other high-performance models. At the same time, our method, with a model size (Size) of 384, maintains a low number of parameters (Params) and computational burden (PLOPs / G) at 27.50M and 13.89G, respectively.In contrast, other high-performance models such as EVA-G/14 and ViT-H/14 showed similar performance in terms of AUC, with values of 90.50% and 89.60%, respectively, but with significantly higher model sizes (336), number of parameters (1013.01M and 632.46M), and computational burdens (445.56G and 363.64G). This indicates that while these models perform well, their complexity and resource requirements limit their practical applicability compared to our method.On the other hand, models like MobileViT-v2 and EfficientNet-v2 performed well in terms of parameter number and computational burden, with parameters of 14.25M and 13.65M, and computational burdens of 12.35G and 8.16G, respectively. However, their lower AUC scores (83.90% and 83.20%) highlight their inferior performance compared to our method in the image classification task.

**Table 3:** Results of comparison experiments on public dataset.

Model	Size	AUC	Params/M	PLOPs/G
EVA-G/14	336	90.50	1013. 01	445. 56
ViT-H/14	336	89.60	632. 46	363. 64
Swin-L	384	88.10	196. 74	100. 28
ResNet-101d	320	84.00	44. 57	23. 82
MobileViT-v2	384	83.90	14. 25	12. 35
EffecientNet-v2	288	83.20	13. 65	8. 16
Ours	384	90.10	27. 50	13. 89

In summary, our method performs well in both comprehensive performance and computational efficiency, making it suitable for image classification tasks that require high performance and low computational burden. The improved model demonstrates significant advantages over larger, high-precision models with similar input sizes, offering notable improvements in both accuracy and computation. Therefore, on the RDD2022 public dataset, our improved model shows excellent accuracy and speed adaptability.

Additionally, we acknowledge the potential limitations or biases in the comparison methods: Performance may vary across different datasets. While we used the RDD2022 dataset, further experiments on other datasets are necessary to validate the generalizability of our model. High-parameter and high-computation models may perform better with ample computational resources. However, in resource-constrained environments, such as mobile or edge devices, our model’s lower parameter count and computational burden offer significant advantages. More complex models like EVA-G/14 and ViT-H/14 may excel in specific tasks but add to training and deployment challenges. Our model strikes a balance by maintaining high performance with lower complexity and computational demand, enhancing its practicality for real-world applications.

**Fig 9:**
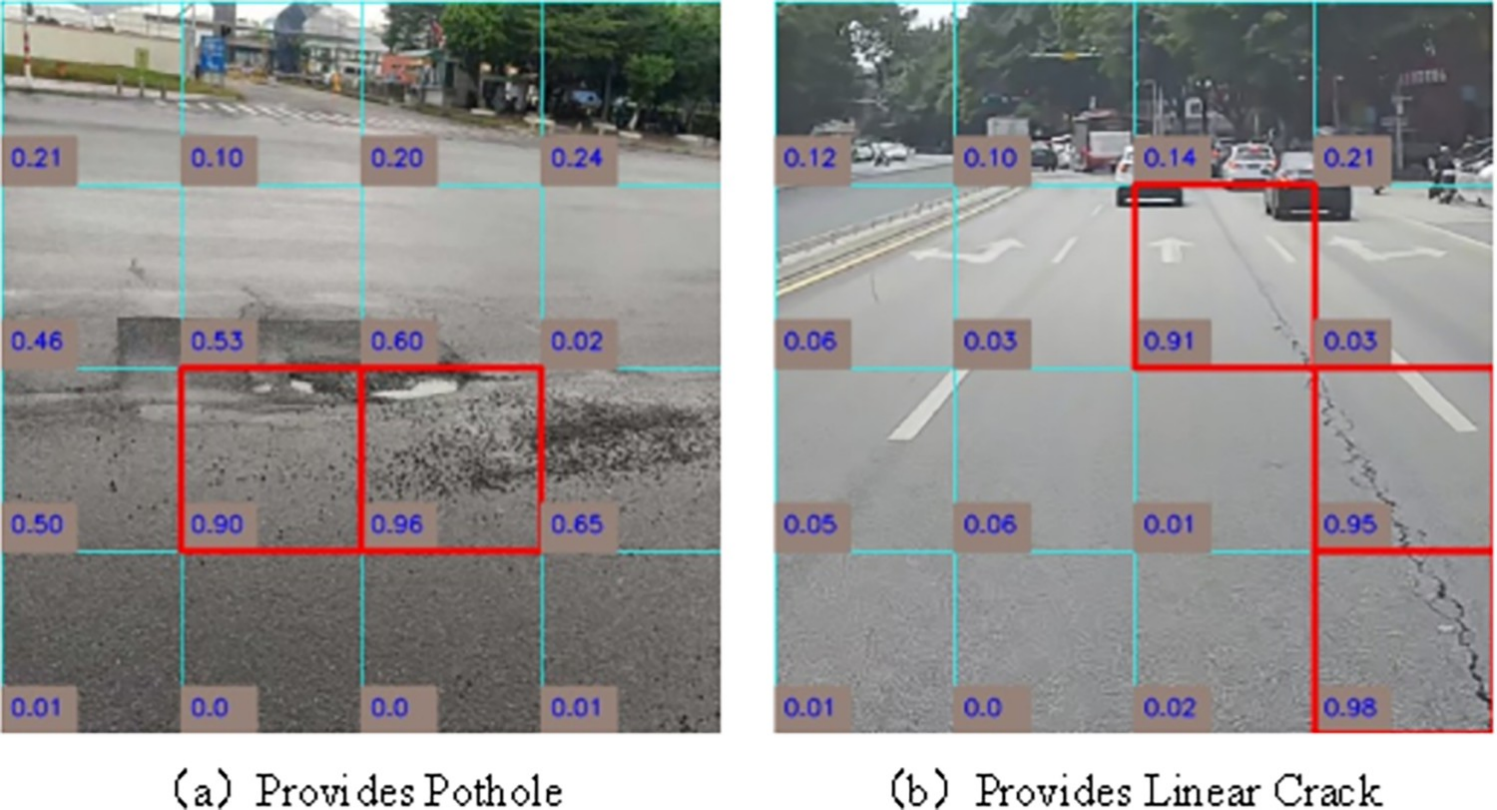
Patch confidence score visualization plot. Republished from Fig 9 under a CC BY 4.0 license, with permission from Feng LIU, original copyright 2024.

LTPLN is detected by judging whether there are small pieces of disease in the image, which is different from the traditional detection mechanism. In this strategy, the labels of the patches in the images are roughly inferred that contain important information to explain and even benefit the resolution of subsequent tasks we visualize the inferred labels with the confidence scores of the patch blocks from the two test images in Fig 9. The observations suggest that the disease patches labels inferred by our approach can further locate disease regions at the disease patch level without any prior positional information for training.

## 6. Discussion

The discussion revolves around the efficacy of the proposed Lightweight Transformer Patch Label Network (LTPLN) for automated pavement damage detection in comparison to traditional methods and other deep learning-based approaches.

Advantages Over Traditional Methods: Traditional road disease detection methods, relying on traditional image processing and hand-crafted features, suffers from poor generalization and difficulty in defining diverse disease features. In contrast, LTPLN, leveraging deep learning and Transformer architecture, shows significant advantages in feature extraction and generalization. By modeling pixel relationships through global operations, LTPLN effectively utilizes image context, compensating for CNNs’ limitations in local operations.

Improvement Over CNN-based Approaches: While CNN-based models like ResNet, DenseNet, and EfficientNet are popular for image classification, they face challenges in handling high-resolution images efficiently. LTPLN addresses this issue by segmenting road images into patches and inferring patch labels using the lightweight Swin Transformer backbone network. This approach not only improves detection accuracy but also meets real-time detection requirements due to its efficient model structure.

Model Efficiency and Adaptability: LTPLN’s lightweight design, incorporating Token Merging and Depthwise Separable Convolution modules, reduces computational burden without compromising accuracy. The Expectation-Maximization Patch Label Distillation strategy further optimizes LTPLN iteratively based on image-level labels, enhancing model adaptability to limited data scenarios. This adaptability is crucial for real-world applications where data availability may be limited.

Comparative Analysis: Comparative experiments with mainstream image classification models demonstrate LTPLN’s superiority in terms of detection accuracy, speed, and computational efficiency. While models like ResNet-34 and DenseNet169 have lower accuracy and efficiency metric, LTPLN achieves a balance between high performance and low computational burden, making it suitable for real-time detection tasks.

Dataset Performance: Evaluation on the RDD2022 dataset showcases LTPLN’s outstanding performance in terms of AUC, model size, and computational burden. Its ability to achieve high accuracy with a relatively lightweight model structure demonstrates its effectiveness in handling complex image classification tasks efficiently.

Future Directions: Future research could focus on further optimizing LTPLN’s architecture for specific road disease detection tasks, exploring additional data augmentation techniques to enhance model robustness, and investigating transfer learning strategies for adapting LTPLN to different road environments.

In conclusion, LTPLN emerges as a promising solution for automated pavement damage detection, offering a balanced performance in terms of accuracy, speed, and computational efficiency, and demonstrating adaptability to real-world data constraints.

## 7. Conclusion

For the problem of poor robustness of automatic pavement detection, a lightweight Transformer patch-based label network (LTPLN) is proposed. LTPLN by inserting Token fusion module to make the model more light, and then use the depth of separable convolution to enhance the classification ability of lightweight Transformer, and then use EM label distillation strategy, only using image label to identify whether the road is disease, and weak supervision iterative training network model can roughly speculate the disease position in the image. Meanwhile, a dataset of road disease detection named GZLD was constructed to evaluate the effectiveness of LTPLN. The experimental results demonstrate the superiority of the present method compared with the mainstream CNN method, and also show that LTPLN is able to locate the disease regions without any prior information about the location. The experimental results show that LTPLN performs excellent in automatic pavement disease detection, the recognition accuracy is close to the current best performance, and the identification and processing speed also meet the real-time requirements. The next step is to continue to collect high-quality road disease datasets to achieve accurate target detection of common road diseases.

Although the proposed Lightweight Transformer Patch Labeling Network (LTPLN) has demonstrated significant performance advantages in automatic pavement disease detection, there are still potential limitations that need further exploration. Firstly, due to the diversity and complexity of pavement diseases, the current model may have limitations in handling specific types or scenarios of diseases. Therefore, future research could explore more fine-grained feature representations and model structure designs to improve the model’s ability to recognize different types of diseases. Secondly, the current model still exhibits some coarseness in locating diseases under weakly supervised learning, necessitating further optimization of the localization algorithm to enhance precision. Additionally, the dataset used in this study may have certain biases, so future work could consider using more comprehensive and realistic datasets for validation and testing.

Future research directions could include but are not limited to: (1) Improving the model’s feature representation and learning capabilities to enhance recognition and localization accuracy of diverse diseases; (2) Exploring the model’s generalization ability across datasets and scenarios to ensure robustness in different environments; (3) Combining advanced data augmentation techniques and transfer learning methods to further improve model performance and generalization; (4) Investigating the integration of pavement disease detection with other domains such as image segmentation, object detection, etc., to explore more application scenarios and possibilities.
